# Identification of an ATP/P2X7/mast cell pathway mediating ozone-induced bronchial hyperresponsiveness

**DOI:** 10.1172/jci.insight.140207

**Published:** 2021-11-08

**Authors:** Xiaomei Kong, William C. Bennett, Corey M. Jania, Kelly D. Chason, Zachary German, Jennifer Adouli, Samuel D. Budney, Brandon T. Oby, Catharina van Heusden, Eduardo R. Lazarowski, Ilona Jaspers, Scott H. Randell, Barry A. Hedgespeth, Glenn Cruse, Xiaoyang Hua, Stephen A. Schworer, Gregory J. Smith, Samir N.P. Kelada, Stephen L. Tilley

**Affiliations:** 1 Department of Respiratory and Critical Care Medicine, The First Hospital of Shanxi Medical University, Taiyuan, China.; 2 Marsico Lung Institute and; 3 Division of Pulmonary Diseases and Critical Care Medicine, Department of Medicine, School of Medicine, University of North Carolina at Chapel Hill, North Carolina, USA.; 4 Wake Forest School of Medicine, Winston-Salem, North Carolina, USA.; 5 Department of Pediatrics and Center for Environmental Medicine, Asthma, and Lung Biology and; 6 Department of Cell Biology and Physiology, School of Medicine, University of North Carolina at Chapel Hill, North Carolina, USA.; 7 Department of Molecular Biomedical Sciences, College of Veterinary Medicine, North Carolina State University, Raleigh, North Carolina, USA.; 8 Department of Head and Neck Surgery & Communication Sciences, Duke University School of Medicine, Durham, North Carolina, USA.; 9 Department of Otolaryngology — Head and Neck Surgery, Carver College of Medicine, University of Iowa, Iowa City, Iowa, USA.; 10 Division of Allergy and Immunology, Department of Pediatrics, and; 11 Department of Genetics, School of Medicine, University of North Carolina at Chapel Hill, Chapel Hill, North Carolina, USA.

**Keywords:** Cell Biology, Immunology, Mast cells

## Abstract

Ozone is a highly reactive environmental pollutant with well-recognized adverse effects on lung health. Bronchial hyperresponsiveness (BHR) is one consequence of ozone exposure, particularly for individuals with underlying lung disease. Our data demonstrated that ozone induced substantial ATP release from human airway epithelia in vitro and into the airways of mice in vivo and that ATP served as a potent inducer of mast cell degranulation and BHR, acting through P2X7 receptors on mast cells. Both mast cell–deficient and P2X7 receptor–deficient (*P2X7^–/–^*) mice demonstrated markedly attenuated BHR to ozone. Reconstitution of mast cell–deficient mice with WT mast cells and *P2X7^–/–^* mast cells restored ozone-induced BHR. Despite equal numbers of mast cells in reconstituted mouse lungs, mice reconstituted with *P2X7^–/–^* mast cells demonstrated significantly less robust BHR than mice reconstituted with WT mast cells. These results support a model where P2X7 on mast cells and other cell types contribute to ozone-induced BHR.

## Introduction

Ozone is a powerful, nonphysiological oxidant produced in major urban areas throughout the world when primary pollutants (nitrogen oxides and volatile organics) from motor vehicle emissions react with sunlight (1). In summer months, ozone levels often exceed the National Ambient Air Quality Standard, contributing to increased morbidity and mortality in patients with cardiovascular and respiratory diseases (2). Long-term ozone exposure is also associated with increased mortality in people with such diseases (3, 4).

Immediate effects of ozone substantially affect the respiratory tract. Inhaled ozone reacts with molecules in the airway surface liquid throughout the upper and lower airways, indirectly stimulating epithelial cells and resulting in well-characterized negative effects on pulmonary function, including acute falls in vital capacity, and more persistent increases in airway resistance and bronchial hyperresponsiveness (BHR) (5–7). These adverse effects on lung physiology occur in individuals without atopic disease and in patients with asthma or chronic obstructive pulmonary disease, potentially resulting in symptom exacerbation and hospitalization for individuals with compromised pulmonary function at baseline.

The mechanisms by which ozone increases airway resistance and induces BHR remain poorly understood. The effect appears independent of ozone-induced neutrophilia but dependent upon products of arachidonic acid metabolism (8, 9). Since ozone exposure results in an increase in mast cell numbers in the bronchial submucosa (10), and mast cells are major producers of arachidonic acid metabolites and other proinflammatory mediators, we hypothesized mast cell activation may be responsible for ozone-induced BHR. In this report, we describe a pathway involving mast cells, ATP, and P2X7 receptors regulating ozone-induced BHR in mice.

## Results

### Ozone increases peripheral airway resistance and induces BHR in mice.

**Figure 1:**
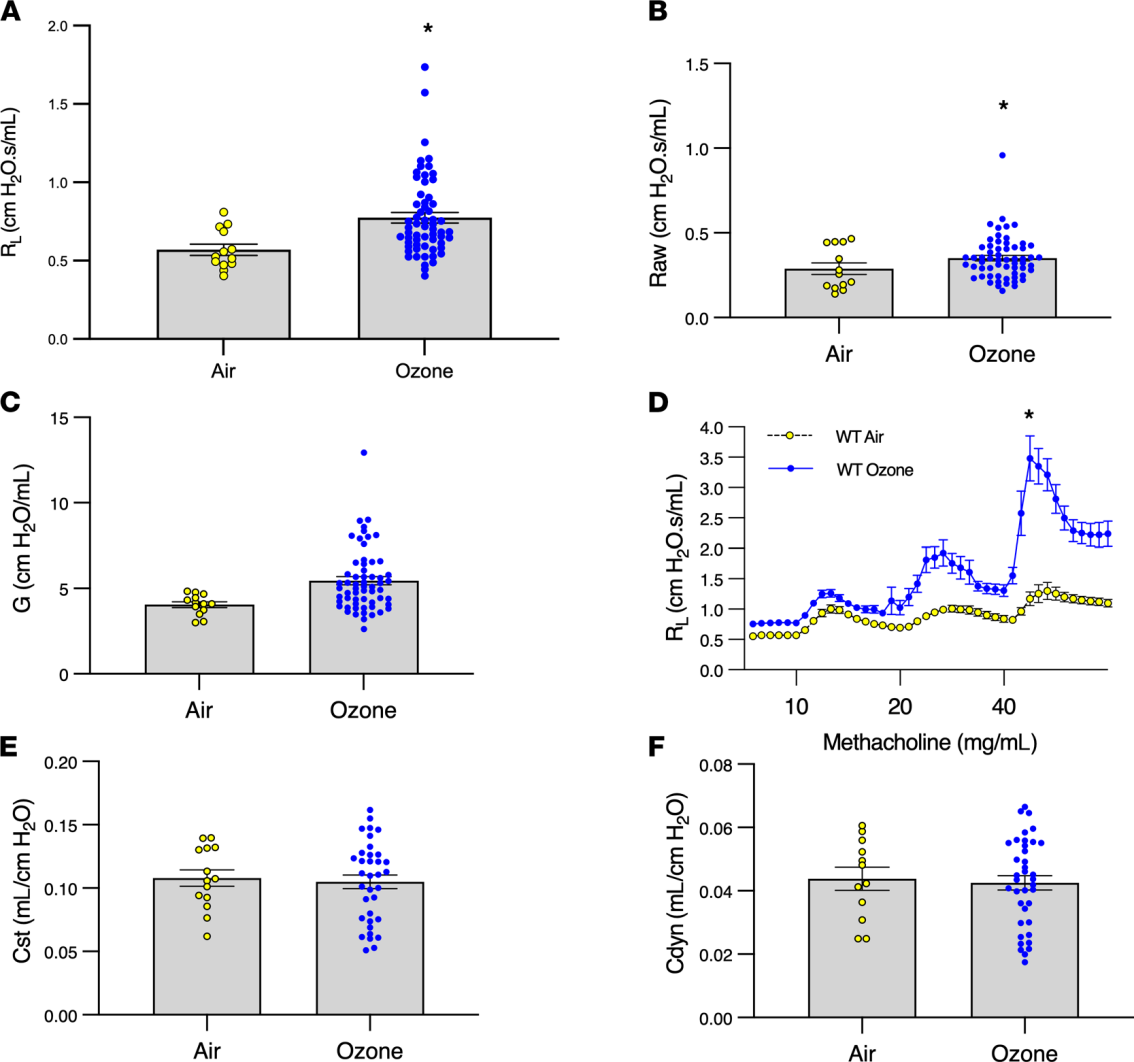
Ozone increases resistance in the murine lung and induces marked BHR in response to methacholine. C57BL/6 WT mice (females, aged 8–40 weeks) were exposed to 2 ppm ozone or air for 3 hours, and airway mechanics at baseline and to graded methacholine challenges were measured 24 hours later for (**A**) total lung resistance (R_L_), (**B**) peripheral airway resistance (G), (**C**) central airway resistance (Raw), (**D**) methacholine challenge, (**E**) static lung compliance (Cst), and (**F**) dynamic lung compliance (Cdyn). Yellow circles represent air-treated mice (*n* = 13), and blue circles represent ozone-treated mice (*n* = 61); *P < 0.05 by Student’s *t* test (**A** and **B**) and mixed effects analysis comparing repeated measures between air and ozone (*P < 0.05) (**D**). Ozone exposure has no effect on static or dynamic lung compliance (**E** and **F**). Data are shown as mean ± SEM.

To determine whether mice develop changes in airway physiology that are similar to humans after ozone exposure, we exposed WT C57BL/6 mice to filtered air or ozone (2 ppm) for 3 hours and measured airway mechanics at baseline and during bronchial challenge with aerosolized methacholine. Overall resistance increased in the murine lung in response to ozone (Figure 1A). A greater effect was seen in peripheral airways (Figure 1B) than in central airways (Figure 1C), similar to what has been reported in humans (6). Overall, the effect of ozone on airway resistance was small and highly variable between mice. In contrast, ozone more consistently induced marked BHR in response to methacholine (Figure 1D). Ozone had no effect on static or dynamic lung compliance (Figure 1, E and F). Collectively, these results showed that ozone produced similar physiological effects in the mouse airway as in humans, supporting the use of this model organism for this investigation.

### Mast cells mediate ozone-induced increases in BHR.

**Figure 2:**
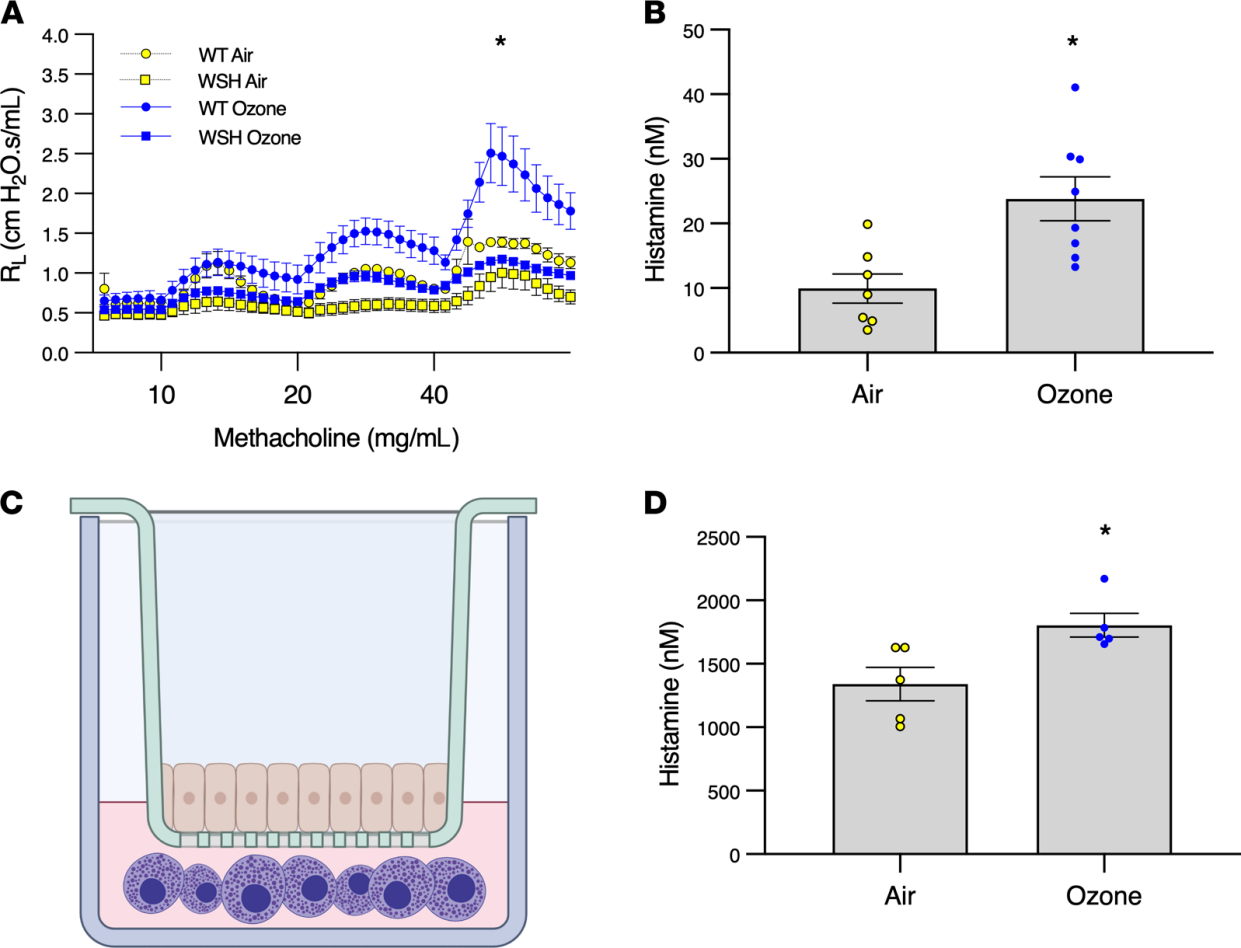
Mast cells mediate ozone-induced BHR, but ozone does not directly activate mast cells. (**A**) Ozone-induced BHR is mast cell dependent. C57BL/6 WT and C57BL/6*Kit^W-sh/W-sh^* mast cell–deficient mice (females, aged 12–30 weeks) were exposed to 2 ppm ozone or air for 3 hours and challenged with methacholine at 24 hours. Yellow circles represent air-treated WT mice (*n* = 3), blue circles represent ozone-treated WT mice (*n* = 14), yellow squares represent air-treated mast cell–deficient mice (*n* = 3), and blue squares represent ozone-treated mast cell–deficient mice (*n* = 14); *P < 0.05 by mixed effects analysis comparing repeated measures between WT and WSH ozone exposures. (**B**) Ozone activates airway mast cells. WT mice (females, aged 10–16 weeks) were exposed to 2 ppm ozone or air for 3 hours and BALF was collected immediately after exposure. Histamine concentrations were measured by ELISA. Yellow circles represent histamine concentrations in BALF from air-treated mice and blue circles represent the same in ozone-treated mice. *n* = 7–8; *P < 0.05 by Student’s *t* test. (**C**) Graphic depicting HBE-ALI/mast cell coculture. (**D**) Ozone indirectly activates mast cells in HBE/mast cell cocultures. Primary HBE cells grown for 21 days under ALI conditions were cocultured with CBMCs and exposed to 0.8 ppm ozone or air for 4 hours at 37°C and 5% CO_2_. Histamine concentrations were measured by ELISA in basolateral media collected immediately after exposure. Yellow circles represent air-treated cells and blue circles represent ozone-treated cells. *n* = 5, *P < 0.05 by Student’s *t* test. Data are shown as mean ± SEM.

To test the hypothesis that ozone-induced changes in BHR are mediated by mast cells, we measured the ability of ozone to increase airway resistance in response to methacholine in *C57BL/6Kit^W-sh/W-sh^* mast cell–deficient mice. Ozone-exposed WT mice demonstrated a significant increase in airway resistance after aerosolized methacholine compared with air-treated controls (Figure 2A). Ozone-exposed mast cell–deficient mice demonstrated more modest increases in resistance after aerosolized methacholine but a statistically significant greater response than air-exposed mast cell–deficient mice. These findings showed mast cells contributed to ozone-induced BHR in mice but also suggest the existence of a concomitant mast cell–independent process.

To demonstrate that ozone activates airway mast cells, histamine was measured in bronchoalveolar lavage fluid (BALF) of WT mice immediately after exposure to ozone. Histamine levels in the airway of ozone-exposed mice were greater than levels measured in air-exposed controls (Figure 2B).

Next, to test whether ozone could directly activate mast cells, human mast cells derived from umbilical cord blood mast cells (CBMCs) and murine bone marrow–derived mast cells (BMMCs) were exposed to ozone or air, and histamine levels in the culture media were measured immediately after exposure. Histamine levels in air-exposed and ozone-exposed CBMCs and BMMCs were similar, indicating ozone did not directly activate mast cells (data not shown). A possible explanation for the discrepancy between our in vivo and in vitro findings is that mast cell activation after ozone exposure occurs indirectly by an epithelial cell–derived mediator(s). To test this hypothesis, the apical side of human bronchial epithelial (HBE) cells cultured at air-liquid interface (ALI) were exposed to ozone or air in the presence of cocultured mast cells in the basolateral compartment (Figure 2C). Greater histamine release was observed from ozone-exposed CBMCs incubated with HBE cells than similar cultures instead exposed to air (Figure 2D). Collectively, these experiments demonstrated that mast cell activation after ozone exposure occurred through an intermediary signaling molecule released by epithelia in response to ozone, and ozone-induced BHR in mice was largely mast cell dependent.

### Ozone stimulates purine release by airway epithelia.

**Figure 3:**
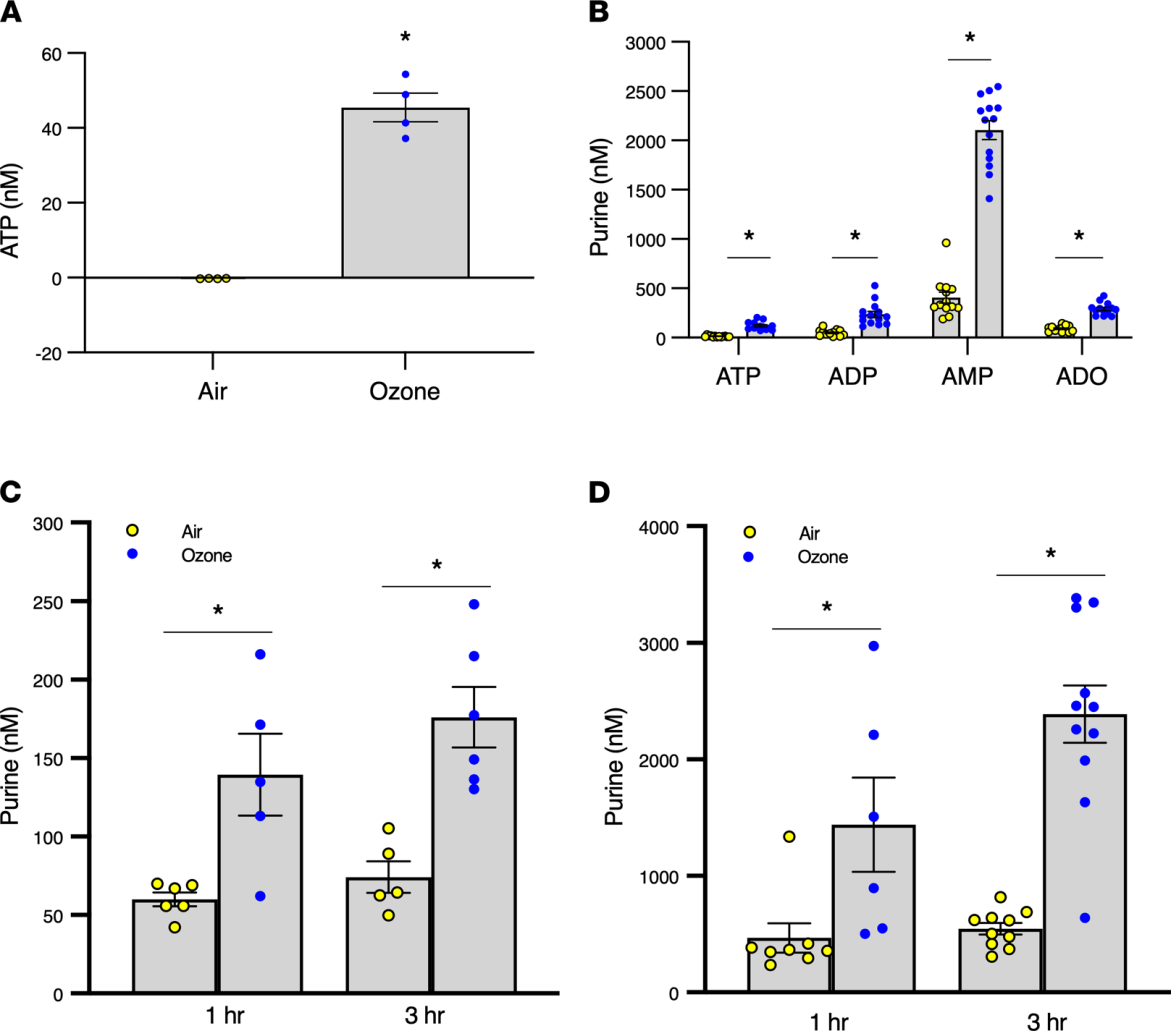
Ozone stimulates ATP release in murine airways and human epithelia in vitro. (**A**) Ozone stimulates ATP release in murine BALF. Mice (females, aged 12–22 weeks) were exposed to 2 ppm ozone or air for 1 hour, and BALF was collected immediately after exposure. ATP was measured via luciferin-luciferase assay. *n* = 4; *P < 0.05 by Student’s *t* test. (**B**) Ozone stimulates nucleotide release in murine BALF. Mice were exposed to 2 ppm ozone or air for 3 hours, and BALF was collected immediately after exposure. Samples were heat-inactivated for 2 minutes and frozen, and purines measured by etheno-derived HPLC. Yellow and blue circles represent nucleotide/side levels from air-treated mice and ozone-treated mice, respectively. *n* = 13–14; *P < 0.05 by Student’s *t* test. (**C**) Ozone stimulates nucleotide release from 16HBE cells. 16HBE epithelial cells were cultured for 28–35 days at ALI and exposed apically to 0.8 ppm ozone or air for 1 hour or 3 hours, and basolateral media was collected immediately after exposure. Purines measured as described in **B**. Data represent total purines (ATP + ADP + AMP + adenosine). Yellow and blue circles represent air- and ozone-treated cells, respectively. *n* = 5–6; *P < 0.05 by 2-way ANOVA with Tukey’s test for multiple comparisons with exposure type (air or ozone) and time as independent variables. (**D**) Ozone stimulates nucleotide release from HBE cells. HBE cells from normal volunteers were obtained, cultured at ALI, and exposed apically to 0.8 ppm ozone or air for 1 hour or 3 hours. Total purines in the basolateral media were measured as described in **B**. Data represent total purines (ATP + ADP + AMP + adenosine). Yellow and blue circles represent air and ozone-treated cells, respectively. *n* = 6–11; *P < 0.05 by 2-way ANOVA with Tukey’s test for multiple comparisons applied with exposure type (air or ozone) and time as independent variables. Data are shown as mean ± SEM.

ATP is an alarmin released from epithelia in response to numerous stimuli (11). Since adenine nucleotides/sides activate mast cells in vitro and in vivo (12–15), we hypothesized ATP and/or its metabolites may be the critical intermediary signaling molecule(s) responsible for ozone-induced mast cell activation. To begin to test this hypothesis, we first investigated and established the capacity of ozone to stimulate ATP release into the airways of mice in vivo and from human epithelial cells in vitro. WT mice were exposed to ozone (2 ppm) or filtered air for 3 hours, and ATP was measured in the BALF immediately after exposure using a luciferin-luciferase assay. ATP levels were 0.25 ± 0.03 nM in BALF from air-exposed mice, but levels increased to 45.5 ± 3.8 nM in ozone-exposed mice (Figure 3A). Since released ATP is rapidly metabolized by cell-surface ectonucleotidases (16, 17), the ATP levels measured above likely underestimate the magnitude of released ATP. Therefore, we performed etheno-derivatization/HPLC analysis to quantify ATP and its catabolites. ATP metabolites were considerably more abundant than ATP in control BALF, but ATP, ADP, adenosine, and to a greater extent AMP were all increased in ozone-treated mouse BALF (Figure 3B).

Although these results illustrated that ozone exposure promoted nucleotide release into the airway lumen, whether ATP release also occurred toward the basolateral compartment cannot be assessed in vivo. Therefore, we used polarized airway epithelial cell culture models to test this possibility. An HBE cell line (16HBE) and primary HBE cells were cultured at ALI and exposed to ozone on the apical side, and purine levels were measured in the basolateral media. Ozone caused substantial accumulation of purines in the basolateral media in both 16HBE and primary HBE cell cultures (Figure 3, C and D). These data suggest ATP and/or one of its metabolites as a candidate intermediary signaling molecule for submucosal mast cell activation in response to ozone.

### ATP activates mast cells and induces BHR.

**Figure 4:**
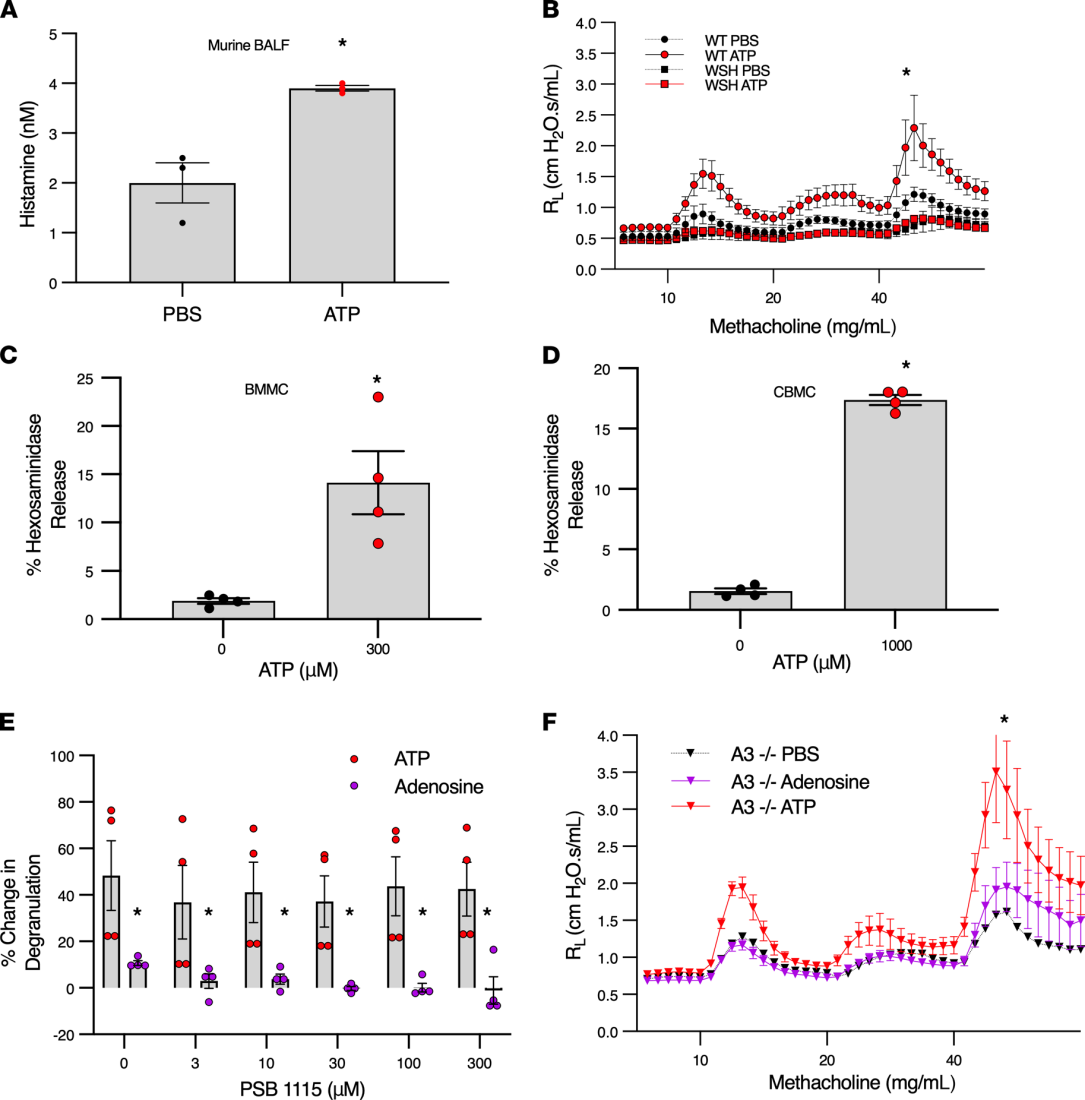
ATP activates mast cells and induces BHR. (**A**) ATP induces murine airway mast cell degranulation. WT mice (females, aged 9–25 weeks) were exposed to aerosolized ATP (50 mg/mL) or PBS; BALF collected after 30 minutes for histamine analysis. Black and red circles represent histamine levels from PBS- and ATP-treated mice, respectively. *n* = 3; *P < 0.05 by Student’s *t* test. (**B**) ATP-induced BHR is mast cell dependent. C57BL/6 WT and C57BL/6Kit^W-sh/W-sh^ mast cell–deficient mice (females, aged 9–25 weeks) were exposed to aerosolized ATP (50 mg/mL), then methacholine 30 minutes later. Black and red circles represent PBS-treated (*n* = 4) and ATP-treated WT mice (*n* = 16), respectively; black and red squares represent PBS-treated (*n* = 3) and ATP-treated mast cell–deficient mice (*n* = 8), respectively; *P < 0.05 by mixed effects analysis between ATP-treated groups. (**C** and **D**) ATP induces degranulation in murine BMMCs (**C**) and human CBMCs (**D**) in vitro. Mast cells were treated with ATP (300 μM, 1000 μM) or PBS for 30 minutes prior to hexosaminidase measurement. Black and red circles represent PBS- and ATP-treated cells, respectively. *n* = 4; *P < 0.05 by Student’s *t* test. (**E**) ATP potentiates antigen-induced degranulation in CBMCs. CBMCs were incubated for 3 days at 1 × 10^6^ cells/mL at 37°C in medium with 10 ng/mL recombinant human IL-4. Cells were incubated with antagonist (PSB1115) or vehicle (DMSO) for 30 minutes prior to ATP or adenosine addition and centrifuged after 30 minutes, and β-hexosaminidase release was assessed. Red and purple circles represent ATP- and adenosine-treated cells, respectively. *n* = 4; *P < 0.05 by Student’s *t* test. (**F**) ATP but not adenosine induces BHR in A_3_*–/–* mice. A_3_*–/–* mice (females, aged 9–25 weeks) were exposed to aerosolized ATP (50 mg/mL), adenosine (50 mg/mL), or PBS and then methacholine 30 minutes later. Purple and red triangles represent adenosine-treated (*n* = 7) and ATP-treated mice (*n* = 8), respectively. *P < 0.05 by mixed effects analysis. Data are shown as mean ± SEM.

To determine the potential of ATP to induce mast cell degranulation and affect airway physiology, we performed a series of experiments in mice, murine BMMCs, and human CBMCs. First, mice were treated with nebulized ATP, and mast cell activation and BHR were evaluated. Like the effect of ozone shown in Figure 2, ATP elicited airway mast cell activation (Figure 4A) and BHR to methacholine (Figure 4B) in WT mice but did not induce BHR in mast cell–deficient mice (Figure 4B). Next, ATP-induced mast cell activation was investigated using murine and human mast cells, which showed ATP directly induced degranulation of murine BMMCs and human CBMCs in culture (Figure 4, C and D). These results are likely due to the direct effect of ATP rather than its metabolite adenosine since we have previously found that adenosine alone does not degranulate BMMCs or CBMCs (15). Adenosine can, however, potentiate antigen-induced degranulation of BMMCs and CBMCs via A_3_ and A_2B_ adenosine receptors, respectively (18, 19). To further establish the role of ATP in mast cell degranulation and exclude adenosine signaling, we tested the ability of ATP to potentiate antigen-induced CBMC degranulation in the presence of a selective A_2B_ adenosine receptor antagonist. Although adenosine receptor antagonism dose-dependently decreased the ability of adenosine to modestly potentiate antigen-induced mast cell degranulation, it had no effect on ATP-induced potentiation of degranulation (Figure 4E). Previously, we showed that adenosine-induced BHR in mice is mediated by the A_3_ adenosine receptor on mast cells (15).

To exclude the possibility that ATP-induced BHR is not due to its metabolite adenosine, we tested the effect of ATP and adenosine in A_3_ adenosine receptor–deficient mice. As expected, and consistent with our previous publication (15), adenosine failed to produce BHR in A_3_ adenosine receptor–deficient mice. In contrast, ATP produced robust BHR in these mice lacking the A_3_ adenosine receptor, indicating catabolism to adenosine is not responsible for ATP-induced BHR (Figure 4F). Collectively, these in vitro and in vivo data support a model in which ozone-induced BHR is mediated by ATP-induced mast cell activation.

### ATP-induced mast cell activation is mediated by the P2X7 receptor.

**Figure 5:**
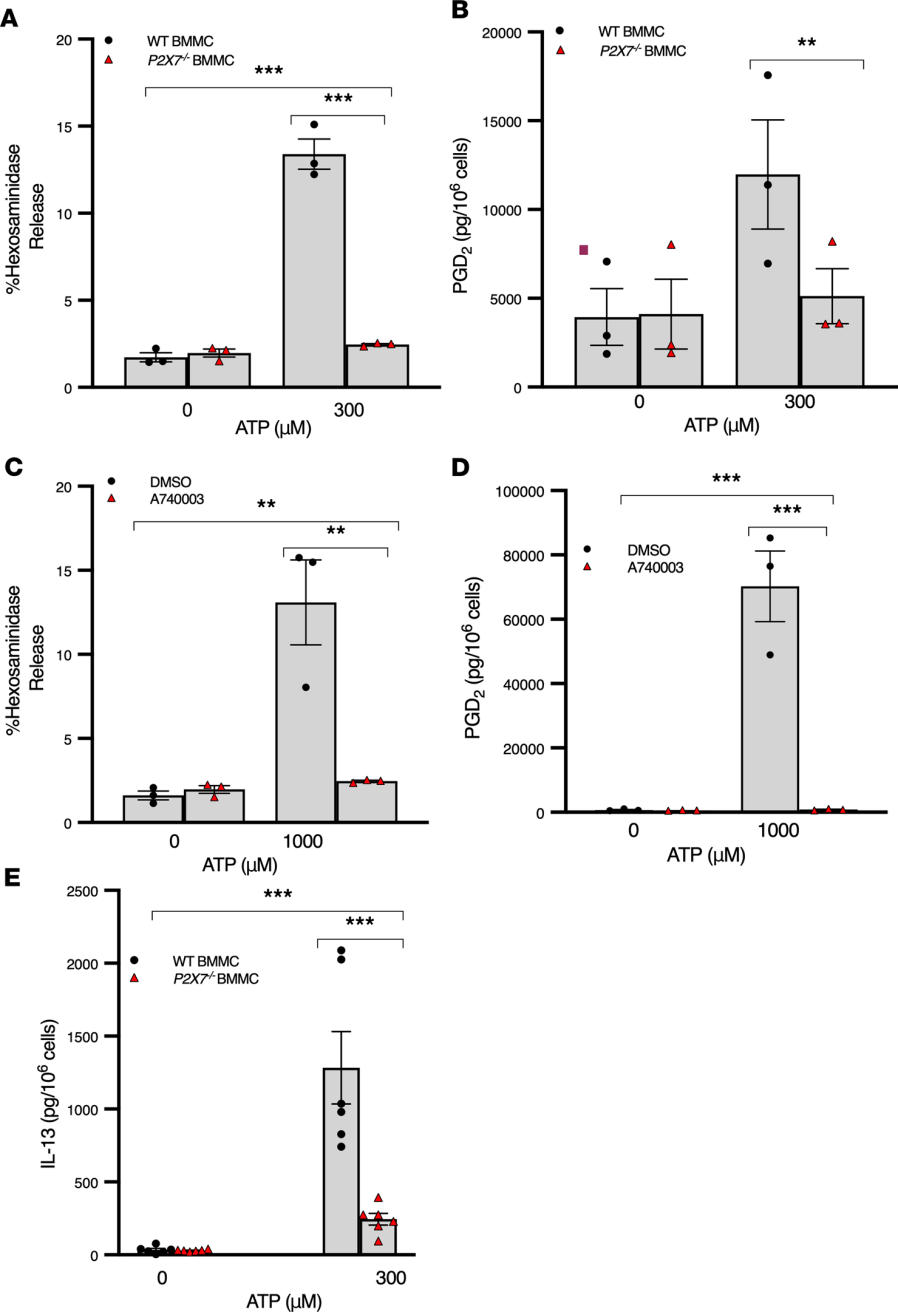
ATP induces mast cell activation via the P2X7 receptor. (**A** and **B**) ATP-induced mast cell degranulation and lipid mediator production is P2X7 dependent. WT and P2X7*–/–* BMMCs were incubated with 0 μM or 300 μM ATP for 30 minutes (**A**) or 4 hours (**B**), and then supernatants were used to measure hexosaminidase release (**A**) or PGD2 concentrations by ELISA (**B**). Black circles represent WT cells; red triangles represent P2X7*–/–* cells. *n* = 2 cell lines per genotype over 3 separate experiments (**C** and **D**) CBMCs incubated 30 minutes with P2X7 antagonist A740003 (3 μM) or vehicle containing equivalent DMSO for 30 minutes prior to 0 μM or 1000 μM ATP for 30 minutes (**C**) or 4 hours (**D**). Black circles represent vehicle treatment; red triangles represent A74003 treatment. *n* = 2 pooled cell lines over 3 separate experiments. (**E**) ATP-induced IL-13 synthesis by BMMCs is attenuated in P2X7*–/–* cells. WT and P2X7*–/–* BMMCs were incubated with 0 μM or 300 μM ATP for 20–24 hours and IL-13 measured in supernatants by ELISA. Black circles represent WT cells; red triangles represent *P2X7^–/–^* cells. *n* = 2 cell lines per genotype over 3 separate experiments (**A**–**E**); **P < 0.005, ***P < 0.001 by 2-way ANOVA with Tukey’s test for multiple comparisons. Data are shown as mean ± SEM.

Extracellular ATP is known to signal through many P2X and P2Y purinergic receptors, variably expressed by different cell types (20). Given that several pharmacological studies have implicated P2X7 as the receptor mediating mast cell degranulation (21, 22), we quantitated mast cell activation in response to ATP in BMMCs from WT mice compared with BMMCs from mice genetically deficient in P2X7 and in CBMCs in the presence or absence of the selective P2X7 receptor antagonist A740003. In WT murine BMMCs and human CBMCs, ATP stimulation resulted in mast cell degranulation and lipid mediator production. These effects of ATP were abolished in P2X7 receptor–deficient BMMCs and in human CBMCs incubated with the P2X7 receptor antagonist A740003 (Figure 5, A–D), despite the expression of other ATP-sensing purinergic receptors by these cells (Supplemental Figure 1; supplemental material available online with this article; https://doi.org/10.1172/jci.insight.140207DS1). In addition, ATP-induced IL-13 synthesis by murine BMMCs was markedly attenuated in P2X7 receptor–deficient cells (Figure 5E). Collectively, these experiments indicate the P2X7 receptor mediated ATP-induced mast cell activation in murine BMMCs and human CBMCs.

### ATP-induced BHR is mediated by P2X7 receptors.

**Figure 6:**
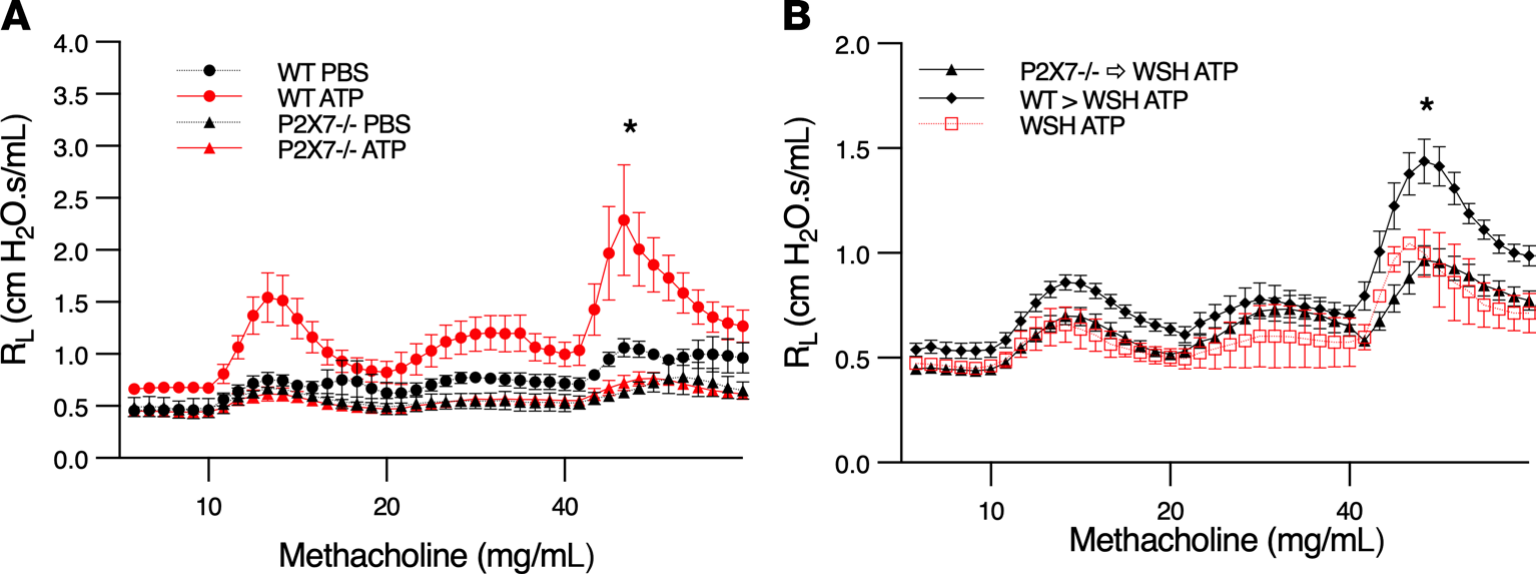
ATP-induced BHR is mediated by P2X7 receptors on mast cells. (**A**) ATP-induced BHR is P2X7 dependent. WT and *P2X7^–/–^* mice (females, aged 9–25 weeks) were exposed to aerosolized ATP (50 mg/mL) and challenged with methacholine 30 minutes later. Black circles represent PBS-treated WT mice (*n* = 4), red circles represent ATP-treated WT mice (*n* = 16), black triangles represent PBS-treated *P2X7^–/–^* mice (*n* = 2), and red triangles represent ATP-treated *P2X7^–/–^* mice (*n* = 4); *P < 0.05 by mixed effects analysis for repeated measures between ATP-treated groups. (**B**) ATP-induced BHR is mediated by P2X7 receptors on mast cells. C57BL/6-*Kit^W-sh/W-sh^* mice (females, aged 4–5 weeks) were reconstituted with either *P2X7^–/–^* or WT BMMCs. After 16 weeks, mice were exposed to aerosolized ATP (50 mg/mL) and challenged with methacholine 30 minutes later. Black diamonds represent mice reconstituted with WT mast cells (*n* = 7), black triangles represent mice reconstituted with *P2X7^–/–^* mast cells (*n* = 7), open red squares represent nonreconstituted C57BL/6-*Kit^W-sh/W-sh^* controls (*n* = 2); *P < 0.05 by mixed effects analysis between reconstituted groups. Data are shown as mean ± SEM.

To determine whether ATP/P2X7 receptor signaling is required for ATP-induced BHR, we treated WT and P2X7 receptor–deficient mice with ATP and measured airway resistance during methacholine challenge. ATP-induced BHR was abolished in P2X7 receptor–deficient mice compared with WT controls (Figure 6A). These results suggest that ATP-induced BHR is entirely dependent upon ATP activation of P2X7 receptor signaling on mast cells.

To rule out the possibility that in vivo mast cell activation by ATP occurs indirectly via an alternative mast cell activator released from another immune cell or structural cell after ATP/P2X7 signaling, we performed reconstitution experiments where mast cell–deficient mice (*C57BL/6Kit^W-sh/W-sh^*) were reconstituted with WT or P2X7 receptor–deficient mast cells to generate animals where P2X7 receptor deficiency was limited to mast cells. Mast cell–deficient mice reconstituted with WT mast cells developed ATP-induced BHR, whereas mice reconstituted with P2X7 receptor–deficient mast cells did not (Figure 6B). These results suggest that ATP triggers mast cell activation directly by stimulating P2X7 receptors on mast cells.

### Ozone-induced BHR is partially mediated by P2X7 receptors on mast cells.

**Figure 7:**
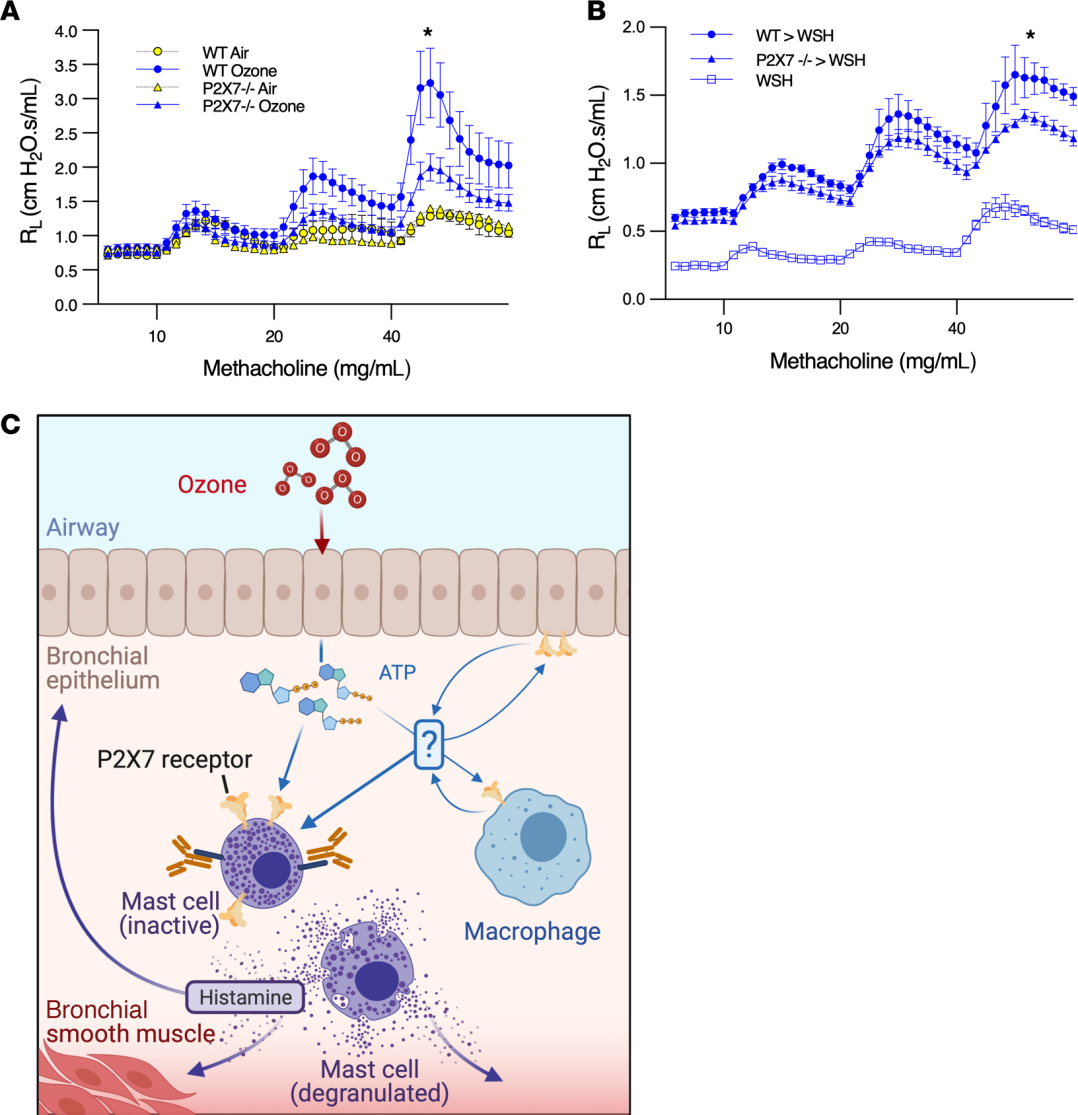
Ozone-induced BHR is partially mediated by P2X7 receptors on mast cells. (**A**) Ozone-induced BHR is partially P2X7 dependent. WT and P2X7*–/–* mice (females, aged 8–16 weeks) were exposed to 2 ppm ozone or air for 3 hours and challenged with 10, 20, or 40 mg/mL methacholine at 24 hours. Yellow circles represent air-treated WT mice (*n* = 3), blue circles represent ozone-treated WT mice (*n* = 13), yellow triangles represent air-treated *P2X7^–/–^* mice (*n* = 1), and blue triangles represent ozone-treated *P2X7^–/–^* mice (*n* = 14); *P < 0.05 by mixed effects analysis between ozone-treated groups. (**B**) Ozone-induced BHR is mediated by P2X7 receptors on mast cells. C57BL/6-Kit^W-sh/W-sh^ mice (females, aged 8 weeks) were reconstituted with either *P2X7^–/–^* or WT BMMCs. After 22 weeks, mice were exposed to 2 ppm ozone or air for 3 hours and challenged with methacholine at 24 hours. Blue circles represent mice reconstituted with WT mast cells (*n* = 5), blue triangles represent mice reconstituted with *P2X7^–/–^* mast cells (*n* = 5), open blue squares represent nonreconstituted C57BL/6-*Kit^W-sh/W-sh^* controls (*n* = 5); *P < 0.05 by repeated-measures 2-way ANOVA between reconstituted groups. (**C**) Illustration depicting ozone/ATP/P2X7/mast cell axis. Data are shown as mean ± SEM.

**Figure 8:**
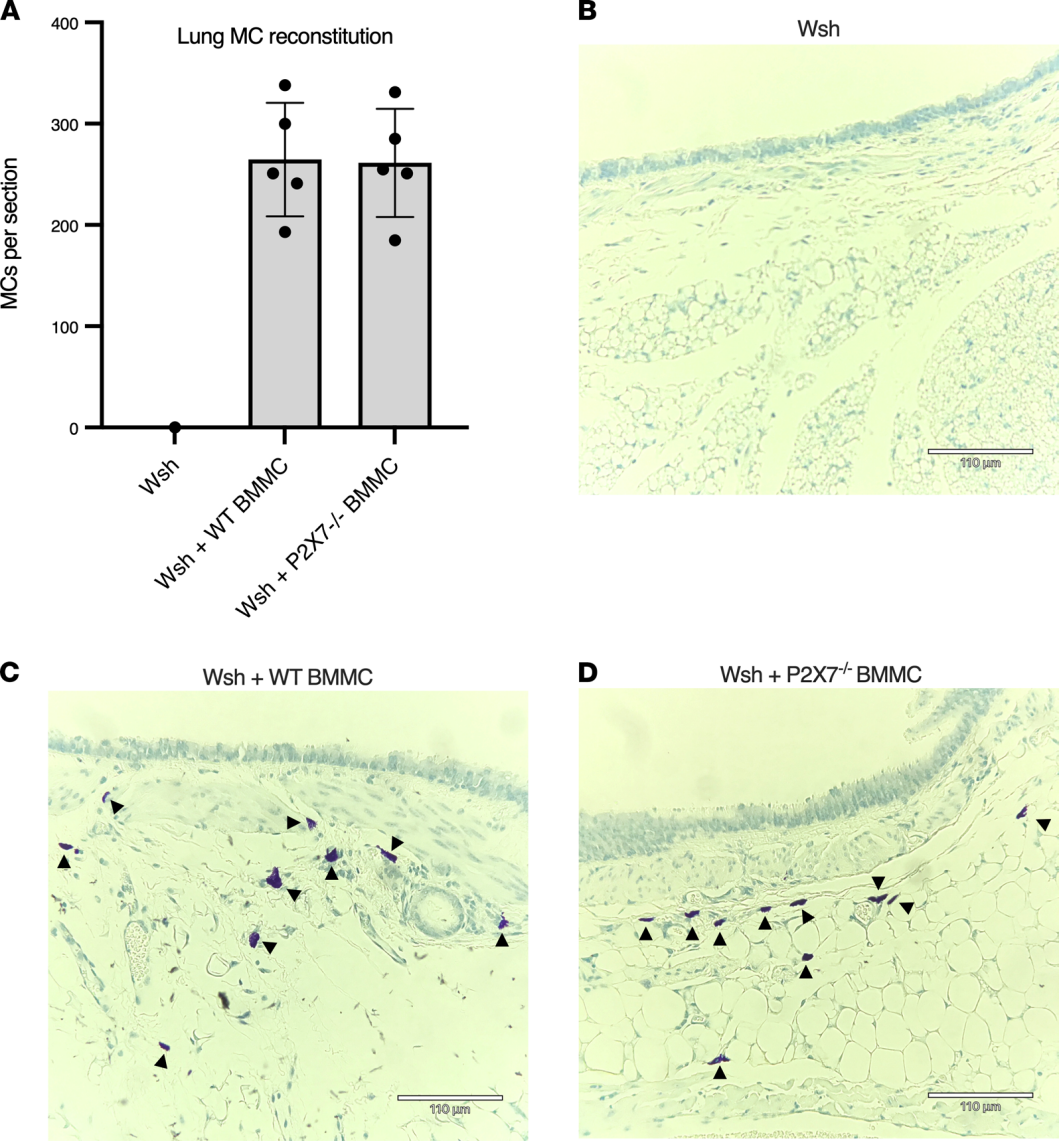
Lung histology of mast cell–reconstituted mice. Immediately after measurement of lung mechanics, lungs from mast cell–reconstituted mice were removed en bloc and fixed in 10% formalin. Paraffin-embedded sections were cut and stained with toluidine blue, and mast cells were counted by an observer blinded to the experimental group. (**A**) Mast cells per section from Wsh mice reconstituted with WT (*n* = 5) and *P2X7^–/–^* (*n* = 5) BMMCs. Data are presented as mean ± SEM. (**B**) Representative section from nonreconstituted Wsh mouse. (**C**) Representative section from Wsh mouse reconstituted with WT BMMCs. (**D**) Representative section from a *Wsh* mouse reconstituted with *P2X7^–/–^* BMMCs. Arrowheads = mast cells. Scale bars: 110 μm.

To determine the contribution of P2X7 receptor signaling to ozone-induced BHR, WT and P2X7-deficient mice were exposed to air or ozone, and airway resistance to methacholine was measured 24 hours later. Although ozone-exposed WT mice demonstrated robust BHR to methacholine, the response was markedly attenuated in mice deficient in P2X7 (Figure 7A). Ozone-exposed P2X7-deficient mice showed a modest increase in resistance compared with air-exposed controls, suggesting a small P2X7-independent component to ozone-induced BHR. Next, to establish that P2X7 receptors on mast cells contribute to ozone-induced BHR, we reconstituted Wsh mast cell–deficient mice with WT and *P2X7^–/–^* BMMCs. BHR induced by ozone was restored in Wsh mice reconstituted with WT mast cells. Wsh mice reconstituted with *P2X7^–/–^* mast cells also regained BHR but to a lesser extent than mice reconstituted with WT cells (Figure 7B). These results suggest that ozone-induced BHR is mediated by P2X7 receptors on mast cells as well as other cell types, as illustrated in Figure 7C. To exclude the possibility that P2X7 deficiency on mast cells affected mast cell reconstitution in the lung, causing the attenuation of BHR in *P2X7^–/–^* reconstituted mice, submucosal and parenchymal mast cell numbers were quantified from reconstituted mice. Mast cell numbers and distribution in the lungs of WT and *P2X7^–/–^* mast cell–reconstituted animals were similar (Figure 8). Taken together, these data support a model in which ozone-mediated BHR is mediated by P2X7 receptors on mast cells and other cell types.

## Discussion

In this report, we identified a critical role for mast cells, ATP, and P2X7 receptors in the development of ozone-induced BHR (Figure 7C). Several previous studies have shown a contribution of mast cells to ozone-induced changes in lung biology (23–25). One of the most comprehensive studies previously investigating the effects of ozone on airway physiology of the mouse was reported by Noviski et al. (26). In this study, WBB6F1-*Kit^W/W-v^* mast cell–deficient mice and WBB6F1*+/+* controls were exposed to ozone or air and specific airway conductance (GL) and dynamic lung compliance (Cdyn) measured in anesthetized tracheostomized mice at baseline and after challenge with i.v. methacholine. A significant difference in Cdyn values of WT mice versus WBB6F1-*Kit^W/W-v^* mast cell–deficient mice was observed 4 hours after 3 ppm ozone exposure, and similar to our studies, no difference in Cdyn was observed after 24 hours. A contributory role of mast cells to ozone-induced BHR was also suggested by the Cdyn response to 1 ppm ozone; however, no contribution was observed at 3 ppm. This discrepancy of reported ozone-induced BHR from the Noviski et al. investigation when compared with our data — wherein a large mast cell contribution to ozone-induced BHR was observed — may be related to differences stemming from the use of i.v. versus aerosolized methacholine or strain-specific differences in the mouse models used. The genetic differences between WBB6F1-*Kit^W/W-v^* mice and C57BL/6-*Kit^W-sh/W-sh^* mice are substantial. WBB6F1*-Kit^W/W-v^* mice have a mutation in the *c-Kit* gene, are on a mixed genetic background, and are infertile and anemic (27). C57BL/6-*Kit^W-sh/W-sh^* mice have an inversion in regulatory elements upstream of c-kit and are on an inbred C57BL6 background (27). Several lines of evidence in humans also support a role for mast cells in mediating the physiological effects of ozone on the airway. Acute ozone exposure causes the release of mast cell–specific mediators into lavageable spaces of the upper and lower airways of human subjects (28, 29). After ozone exposure, mast cell numbers in bronchial mucosa increased 2-fold in normal subjects (30), but in asthmatic humans, a 4-fold increase was observed despite treatment with inhaled corticosteroids (10).

Mast cell activation by ozone appears to occur indirectly. Peden et al. exposed a rat mast cell line to ozone and found no evidence of mast cell degranulation (31). Consistent with these reports, our data also showed that isolated cultures of human and murine mast cells did not degranulate after direct exposure to ozone; however, degranulation was observed when cocultured with epithelia. These data suggest an epithelial-derived mediator, released by exposure to ozone, triggers mast cell degranulation. Several lines of evidence suggest ATP is such a mediator. Extracellular ATP and its metabolites are well-recognized signaling molecules. Epithelia release ATP via conductive pathways mediated by pannexin-1 channels (32), and via exocytosis of granules storing ATP via vesicular nucleotide transport (33, 34). Our data demonstrated substantial ATP release occurred from airway epithelia in vivo and in vitro in response to ozone. Based on the robust ecto-ATPase activity present in airway epithelia (16, 17), rapid metabolism of released ATP likely accounted for the relative distribution of purines (AMP >>ADP/ADO>ATP) we observed in extracellular samples. The presence of elevated purine levels on the basolateral side of HBE-ALI cultures supports the hypothesis that ozone-induced epithelial ATP release activates mast cells in the submucosa. Indeed, coculture experiments showing mast cell degranulation after HBE cell exposure to ozone further supports this hypothesis.

Since ATP released by epithelia is rapidly degraded into adenosine, which activates mast cells (13, 14, 35), we first hypothesized that adenosine rather than ATP was responsible for mast cell degranulation after ozone exposure. In mice, adenosine activates mast cells in the airway via the A_3_ adenosine receptor (36); however, ATP-mediated BHR was robust in mice lacking the A_3_ receptor, similar to WT controls. These results suggest that ATP, rather than adenosine, is responsible for ozone-induced mast cell activation. To further substantiate this conclusion, we tested the ability of adenosine and ATP to degranulate human mast cells in vitro. ATP was much more potent than adenosine at triggering mast cell degranulation. To exclude any effects of metabolism of ATP to adenosine, we treated cells with a selective A_2B_ receptor antagonist, the adenosine receptor implicated in the degranulation of human mast cells, to eliminate adenosine-induced mast cell activation (19). Although adenosine-induced mast cell degranulation was abolished in the presence of A_2B_ antagonist, ATP-induced degranulation remained intact.

Extracellular ATP activates cells through many purinergic receptors. Our in vivo observations showing attenuation of ozone- and ATP-induced BHR in P2X7-deficient mice suggest that the P2X7 receptor on mast cells is involved. Previous pharmacological studies have suggested roles for P2X and P2Y receptors in mediating ATP-induced mast cell activation (37). Although early studies were limited because of lack of specificity of ligands used, more recent studies with selective receptor antagonists have shown evidence for functional P2X1, P2X4, and P2X7 receptors on human lung mast cells and the LAD2 human mast cell line, and similar to our findings with CBMCs, ATP induces degranulation of LAD2 cells via P2X7 (21, 38). Our data with mast cells cultured from P2X7 receptor–deficient mice are consistent with pharmacological studies suggesting that P2X7 receptors mediate degranulation of murine BMMCs (39).

Marked attenuation of BHR in mast cell–deficient mice and in P2X7-deficient mice initially suggested the possibility that direct activation of P2X7 receptors on mast cells played a major mechanistic role in the development of ozone-induced BHR. However, while such a pathway appears to be contributory, substantial restoration of the BHR response was still present in mast cell–deficient mice reconstituted with *P2X7^–/–^* mast cells. These results suggest that P2X7 receptor activation on other cell types contributes substantially to ozone-induced mast cell activation. P2X7 receptors demonstrate a broad tissue distribution and are expressed by airway epithelia and most immune cells. Activation of P2X7 results in production and secretion of mature IL-1β and IL-18 through the activation of the NLRP3 inflammasome, and both cytokines can directly activate mast cells (40, 41). Our results also showed a small P2X7-independent component to ozone-induced BHR in mice. Ozone stimulates the release of several mediators from epithelia capable of activating mast cells, including IL-33, and synergistic signaling of mast cells by ATP and IL-33 has been reported (42, 43).

Presently, there exists no recognized therapeutic intervention to prevent the adverse effects of ozone on susceptible populations, such as patients with atopy or chronic lung disease. Continued investigations on the role that ATP, P2X7 receptors, and mast cells play in humans, and strategies targeting this pathway, may lead to preventive approaches for reducing morbidity and mortality from ozone.

## Methods

### Animals.

C57BL/6-*Kit^W-sh/W-sh^* mast cell–deficient mice, *P2X7^–/–^* mice, and WT controls, all on the C57BL/6 genetic background, were bred in a pathogen-free facility on a 12-hour light/12-hour dark cycle on the University of North Carolina (UNC) campus. For all experiments, mice were older than 8 weeks and female.

### Ozone exposure of mice.

Mice were placed in individual wire mesh cages within a stainless steel and glass chamber and exposed to 2 ppm ozone in air or alone for 3 hours. An ozone generator (Yanco) using UV light produced ozone from filtered air that was pumped into the exposure chamber at 10 L/min. Ozone and air were circulated within the exposure chamber using a fan, and ozone levels were assessed in real time using an ozone analyzer (OzoneSolutions) detecting a range from 0.2 to 5 ppm ozone and as previously described (44, 45).

For nucleotide measurements, mice were anesthetized and gently lavaged with 0.6 mL HBSS (Gibco) immediately after ozone exposure, and BALF samples were frozen for subsequent purine measurement.

For airway physiology assessment, 24 hours after exposure, mice were anesthetized for the measurement of baseline airway resistance and airway responsiveness to inhaled aerosolized methacholine. BALF was collected and supernatants were frozen for histamine measurements.

### Administration of ATP to mouse lungs.

Mice were given 50 mg/mL of ATP (MilliporeSigma) or PBS (Gibco) aerosolized into a plexiglass full-body exposure chamber (Buxco Systems) for 10 minutes. Mice were anesthetized 30 minutes later for measurement of airway mechanics. BALF was collected and supernatant was frozen for histamine measurements.

### Airway physiology.

Mice were anesthetized with pentobarbital, tracheostomized, and paralyzed with atracurium. Mice were then mechanically ventilated with a computer-controlled ventilator (Scireq) at 300 breaths/min, with a tidal volume of 6 cc/kg and a PEEP of 3 cm. Baseline airway resistance and bronchial responsiveness to inhaled aerosolized methacholine were assessed with 10-second challenges of 10, 20, and 40 mg/mL methacholine (MilliporeSigma) as previously described (46). Physiological assessments included total airway resistance (R_L_), central airway resistance (R_aw_), peripheral airway resistance (G), dynamic lung compliance (C_dyn_), and static compliance (C_st_).

### BMMC culture.

Bone marrow from C57BL/6 WT and C57BL/6 *P2X7^–/–^* mice was extracted and cultured in IMDM (Gibco) supplemented with 10% FBS (Gibco), 1 mM sodium pyruvate (Cellgro), 10 mM HEPES (Cellgro), MEM NEAA (Gibco), penicillin-streptomycin (Gibco), 50 μM β-mercaptoethanol (MilliporeSigma), 20 ng/mL recombinant murine (rm) stem cell factor (SCF) (Peprotech), and 10 ng/mL rmIL-3 (MilliporeSigma). Cells were cultured at 37°C and 5% CO_2_ for at least 6 weeks at 1 × 10^6^ cells/mL. Cells at 6 weeks old were determined to be more than 95% mast cells by Kimura stain.

### CBMC culture.

Heparinized cord blood was obtained from the Carolinas Cord Blood Bank at UNC and Duke hospitals. Cells were cultured as previously described (19).

### 16HBE culture.

16HBE14o (16HBE) cells, an SV40 plasmid transformed HBE cell line, were obtained in-house. 16HBE cells were plated on fibronectin-coated Millicell inserts (0.4 μm, 12 mm; MilliporeSigma) and grown in MEM (Gibco) supplemented with 10% FBS (Gibco), penicillin-streptomycin (Gibco), and 1% L-glutamine (Life Technologies) for 1 week until confluent. Apical media was removed, and cells were grown for 1 day at ALI at 37°C and 5% CO_2_ prior to air/ozone exposures.

### HBE culture.

HBE cells from previously healthy organ donors whose lungs were unsuitable for transplant were obtained from the Marsico Lung Institute Tissue Procurement and Cell Culture Core (UNC at Chapel Hill) and cultured using procedures and ALI growth medium previously described in detail previously (47). HBE cells were plated on human placental collagen type IV–coated (MilliporeSigma) Millicell inserts (0.4 μm, 12 mm; MilliporeSigma) at a density of 2.5 × 10^5^ cells/insert. Once confluent, the growth medium was removed from the apical side and cells were differentiated at ALI for at least 21 days at 37°C and 5% CO_2_. Cultures were washed with PBS (Gibco) and basolateral ALI growth medium was replaced 3 times per week.

### Ozone exposure in cells.

All cells were exposed to ozone in chambers operated by the US Environmental Protection Agency and exposed to 0.8 ppm ozone or air for 0.5–4 hours at 37°C and 5% CO_2_.

Before exposure, 16HBEs and primary HBE cells were gently washed 3 times with PBS and Millicells were placed in 12-well plates over 500 μL HBSS (Gibco) per well. Cells were exposed to 0.8 ppm ozone or air for 0.5, 1, or 3 hours. Immediately after exposure, basolateral HBSS was collected for nucleotide measurement or histamine assay.

In CBMC and HBE cell cocultures, CBMCs were incubated for 3 days at 1 × 10^6^ cells/mL in medium at 37°C and 5% CO_2_ with 10 ng/mL recombinant human IL-4 (Invitrogen). Before exposure, cells were washed and resuspended at 1 × 10^6^ cells/mL in ALI media with 100 ng/mL recombinant human (rh) SCF (Life Technologies) and 50 ng/mL rhIL-6 (Life Technologies). HBE cells were cultured at ALI on Millicells for at least 21 days. Cells were gently washed 3 times with PBS, and Millicells were placed in 12-well plates over 500 μL CBMCs in ALI media per well. Cells were exposed to 0.8 ppm ozone or air for 0.5, 1, or 3 hours. Immediately after exposure, basolateral media was collected for nucleotide measurement or histamine assay.

### Quantification of ATP by the luciferin luciferase assay.

ATP levels were quantified via a GloMax luminometer (Promega) as previously described (48). Briefly, sample aliquots (20 μL) were transferred to a 96-well plate containing 150 μL H_2_O/well and placed in the luminometer chamber. Seventy-five microliters of the luciferin-luciferase buffer were subsequently injected, and luminescence was read and integrated every 4 seconds using the GloMax Discover software. ATP calibration curves were performed in parallel to samples.

### Etheno-derivatization and HPLC analysis of nucleotides.

All samples collected for nucleotide measurement were heat-inactivated for 2 minutes and frozen, and purines were measured by etheno-derivatization HPLC as previously described (49). Briefly, samples were incubated at 80°C for 40 minutes with 0.5 M 2Cl-acetaldehyde in citrate-phosphate etheno-derivatization buffer at pH 4.0. Etheno-derived products were separated by HPLC using a Chromolith column (Merck).

### P2 receptor expression.

Total RNA was extracted from CBMCs incubated 3 days with 10 ng/mL recombinant human IL-4 (Invitrogen) and from BMMCs with RNA-bee (Tel-Test). RNA was treated with RQ1 DNAse (Promega) and reverse-transcribed using the High-Capacity cDNA Archive Kit (Applied Biosystems). Gene expression relative to controls was determined by real-time PCR using the Universal Master Mix (Applied Biosystems) and the following gene expression assays (Applied Biosystems) (Hs, human; Mm, mouse): P2X1: Hs00175686_m1, Mm00435460_m1; P2X2: Hs00247255_m1, Mm00462952_m1; P2X3: Hs00175689_m1, Mm00523699_m1; P2X4: Hs00602442_m1, Mm00501787_m1; P2X5: Hs00531938_m1, Mm00473677_m1;P2X6: Hs01003997_m1, Mm00440591_m1; P2X7: Hs00175721_m1, Mm00440578_m1; P2Y1: Hs00704965_s1, Mm00435471_m1; P2Y2: Hs00175732_m1, Mm02619978_s1; P2Y4: Hs04176265_s1, Mm00445136_s1; P2Y6: Hs00366312_m1, Mm01275473_m1; P2Y11: Hs01038858_m1 (nonexistent in mouse); P2Y12: Hs00224470_m1, Mm01950543_s1; P2Y13: Hs00256749_s1, Mm00546978_m1; P2Y14: Hs04176258_s1, Mm01289602_m1; GAPDH: Hs99999905_m1; β-actin: Mm00607939_s1.

Gene levels were determined as percentage expression of housekeeping gene (GAPDH in humans, β-actin in mice) by the formula 2^–ΔCt^ × 100.

### β-hexosaminidase release.

CBMCs were incubated for 3 days at 1 × 10^6^ cells/mL at 37°C in medium with 10 ng/mL recombinant human IL-4 (Invitrogen); BMMCs were untreated. After resuspension in Siraganian buffer, 1 × 10^5^ cells were transferred to wells of 96-well microtiter plates. In experiments using P2X7 antagonist A740003 (Tocris), cells were incubated with antagonist or vehicle (DMSO) in Siraganian buffer for 30 minutes prior to ATP addition. In experiments using A2B antagonist PSB1115 (Tocris), cells were incubated with antagonist or vehicle (DMSO) in Siraganian buffer for 30 minutes prior to ATP or adenosine addition. ATP or adenosine dissolved in sterile ultrapure water, adjusted to pH 7.1 with NaOH, was added and cells incubated for 30 minutes. Reactions were terminated by incubating cells on ice and centrifuging at 350*g* for 10 minutes at 4°C. The extent of mast cell degranulation was determined by comparing β-hexosaminidase activity in the supernatant and cell pellets lysed with 0.1% Triton X-100. Next, β-hexosaminidase activity was determined by incubating supernatant and cell lysate with 1 mM *p*-nitrophenyl-*N*-acetyl-β-d-glucosamine (MilliporeSigma) in citrate buffer (0.1 M citric acid, 0.1 M sodium citrate, pH 4.5) for 1 hour at 37°C. The reaction was terminated by adding 0.1 M Na_2_CO_3_/NaHCO_3_, and the absorbance was measured at 405 nm. The β-hexosaminidase release was expressed as a percentage of the total amount of β-hexosaminidase present in the cells (50).

### Cytokine and PGD2 release.

CBMCs were incubated for 3 days at 1 × 10^6^ cells/mL in medium at 37°C with 10 ng/mL recombinant human IL-4 (Invitrogen); BMMCs were untreated prior to assay. Cells were resuspended in fresh medium; rhIL-4 was included for human experiments. In experiments using P2X7 antagonist A740003 (Tocris), cells were incubated with 3 μM A740003 or vehicle (DMSO) in medium for 30 minutes prior to ATP addition. ATP dissolved in sterile ultrapure water and adjusted to pH 7.1 with NaOH or vehicle was applied and cells incubated for 4 hours (PGD2) or 20–24 hours (cytokines). Cells were pelleted and supernatants assayed by ELISA for PGD2 (Cayman Chemical) or IL-13 (eBioscience).

### Histamine analysis.

Cells were pelleted and supernatants assayed by ELISA for histamine (Cayman Chemical) according to kit instructions.

### Mast cell reconstitution.

BMMCs were cultured as described above. BMMCs were centrifuged at 300*g* at 4°C for 10 minutes and resuspended in PBS at a concentration of 10 million cells/200 μL. C57BL/6-*Kit^W-sh/W-sh^* recipient mice, 4–5 weeks old, were injected with 10 million cells via the tail vein. After 14–16 weeks, animals were exposed to ATP or 2 ppm ozone for 3 hours, and BHR was assessed as described above.

### Statistics.

Statistical analysis was performed using GraphPad Prism. An unpaired 2-tailed Student’s *t* test was used when comparing 2 groups; 2-way ANOVA with Tukey’s test for multiple comparisons when comparing more than 2 groups with 2 independent variables was used for the determination of statistically significant differences, as specified in the figure legends; *P* less than 0.05 was considered significant. For repeated measures (mixed effects analysis denoted in figure legends), we analyzed data by fitting a mixed model as implemented in GraphPad Prism because repeated measures ANOVA cannot handle missing values. This model uses a compound symmetry covariance matrix and is fit using restricted maximum likelihood. In the absence of missing values, this method gives the same *P* values and multiple-comparison tests as repeated measures ANOVA. In the presence of missing values (missing completely at random), the results can be interpreted like repeated measures ANOVA.

### Study approval.

All studies were conducted in accordance with the IACUC guidelines of UNC at Chapel Hill. Heparinized cord blood samples from the Carolinas Cord Blood Bank at UNC and Duke hospitals were deidentified units with volumes or cell numbers too low for use by the Carolinas Cord Blood Bank, and their use for this research was determined to be exempt from IRB approval. HBE cells were procured and utilized as described above under a protocol approved by the Committee on the Protection of the Rights of Human Subjects at UNC at Chapel Hill.

## Author contributions

XK, WCB, CMJ, ZG, JA, ERL, and SLT designed experiments. XK, WCB, CMJ, KDC, ZG, JA, SDB, BTO, CVH, BAH, GC, GJS, SNPK, ERL, and SLT performed experiments and analyzed data. XK, WCB, CMJ, SAS, XH, and SLT wrote the manuscript. IJ, SHR, and SNPK provided reagents and expertise on experimental design, performance, and interpretation. SAS provided statistical analysis. All authors reviewed and approved the manuscript. Order of co–first authors was based on the length of time spent on the project.

## Supplementary Material

Supplemental data

## Version 1 09/21/2021

In-Press Preview

## Version 2 11/08/2021

Electronic publication
