# Supplementary figures and images for: Identification of an ATP/P2X7/mast cell pathway mediating ozone-induced bronchial hyperresponsiveness
Source: JCI Insight. 2021 Nov 8;6(21):e140207. doi: 10.1172/jci.insight.140207 (PMC8663556; doi:10.1172/jci.insight.140207)

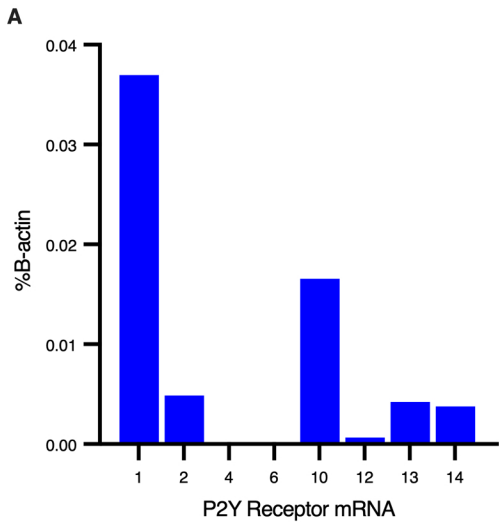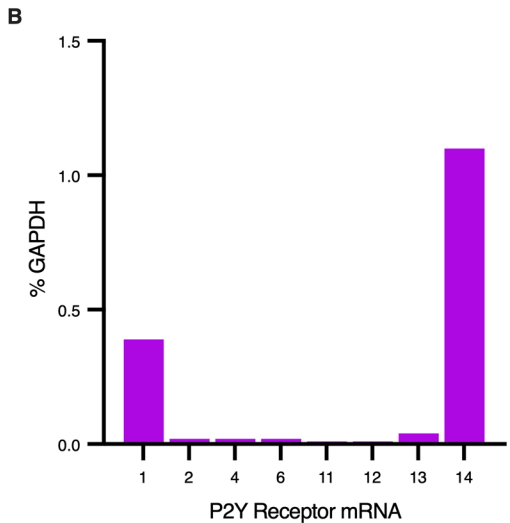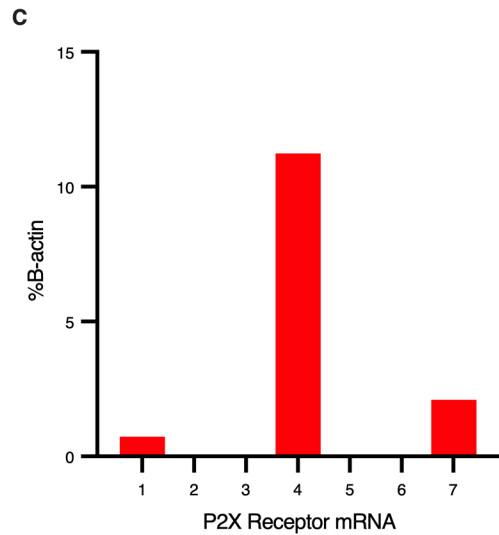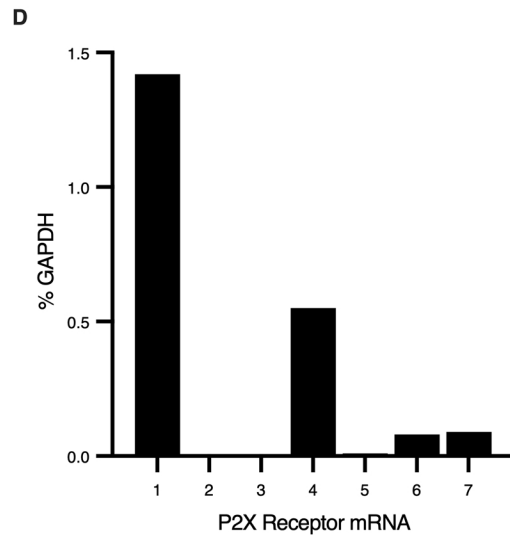

Supplement: Supplemental data [file jciinsight-6-140207-s098.pdf]
